# Genetic dissection of TMEM106B in tauopathy

**DOI:** 10.1002/alz70861_108381

**Published:** 2025-12-23

**Authors:** Joanna L Jankowsky

**Affiliations:** ^1^ Baylor College of Medicine, Houston, TX USA

## Abstract

**Background:**

TMEM106B is a risk modifier of multiple neurological conditions, where a single coding variant and multiple non‐coding SNPs influence the balance between susceptibility and resilience. Two key questions that emerge from past work are whether the lone T185S coding variant contributes to protection, and if the presence of TMEM106B is helpful or harmful in the context of disease. Here, we address both questions while expanding the scope of TMEM106B study from TDP‐43 to models of tauopathy.

**Method:**

We generated knockout mice with constitutive deletion of TMEM106B, alongside knockin mice encoding the T186S knock‐in mutation (equivalent to the human T185S variant), and crossed both with a P301S transgenic tau model to study how these manipulations impacted disease phenotypes.

**Result:**

We found that TMEM106B deletion accelerated cognitive decline, hindlimb paralysis, tau pathology, and neurodegeneration. TMEM106B deletion also increased transcriptional correlation with human AD and the functional pathways enriched in KO:tau mice aligned with those of AD. In contrast, the coding variant protected against tau‐associated cognitive decline, synaptic impairment, neurodegeneration, and paralysis without affecting tau pathology. We further identify a bidirectional effect on lysosomal number in microglia of tau mice upon TMEM106B manipulation. Experiments in primary microglia demonstrate that loss of TMEM106B has no impact on tau PFF update, but selectively impairs degradation.

**Conclusion:**

Our findings reveal that TMEM106B is a critical safeguard against tau accumulation, and that loss of this protein has a profound effect on sequelae of tauopathy. Our study further demonstrates that the coding variant is functionally relevant and contributes to neuroprotection downstream of tau pathology to preserve cognitive function.

